# Electronic Versus Traditional Data Collection: A Multicenter Randomized Controlled Perioperative Pain Trial

**DOI:** 10.1080/24740527.2019.1587584

**Published:** 2019-07-30

**Authors:** James S. Khan, Lindsay A. Jibb, Jason W. Busse, Ian Gilron, Stephen Choi, James E. Paul, Michael McGillion, Sean Mackey, D. Norman Buckley, Shun Fu Lee, P. J. Devereaux

**Affiliations:** a Department of Anesthesia, Mount Sinai Hospital, University of Toronto, Toronto, Ontario, Canada; b School of Nursing, Faculty of Health Sciences, University of Ottawa, Ottawa, Ontario, Canada; c Evidence-to-Practice Program, Children’s Hospital of Eastern Ontario Research Institute, Ottawa, Ontario, Canada; d Department of Anesthesia, McMaster University, Hamilton, Ontario, Canada; e Department of Health Research Methods, Evidence, and Impact, McMaster University, Hamilton, Ontario, Canada; f Michael G. DeGroote Institute for Pain Research and Care, McMaster University, Hamilton, Ontario, Canada; g The Michael G. DeGroote Centre for Medicinal Cannabis Research, McMaster University, Hamilton, Ontario, Canada; h Department of Anesthesiology & Perioperative Medicine, Queen’s University, Kingston, Ontario, Canada; i Department of Anesthesia, Sunnybrook Health Sciences Center, Toronto, Ontario, Canada; j Population Health Research Institute, McMaster University, Hamilton, Ontario, Canada; k School of Nursing, Faculty of Health Sciences, McMaster University, Hamilton, Ontario, Canada; l Division of Pain Medicine, Department of Anesthesiology, Perioperative, and Pain Medicine, Stanford University, Palo Alto, CA, USA; m Department of Medicine, McMaster University, Hamilton, Ontario, Canada

**Keywords:** pain, perioperative, clinical, clinical trials, randomized controlled trials

## Abstract

**Background**: Electronic data collection is increasingly available as a means to collect pain-related clinical trial data; however, effectiveness and costs relative to traditional data collection are uncertain.

**Aims**: The aim of this study was to evaluate data quality, protocol adherence, satisfaction, and resource requirements of electronic data collection (i.e., Internet-based electronic submission) compared to traditional data collection methods (i.e., paper-based diaries and telephone interviews) in a perioperative factorial randomized controlled trial.

**Methods**: This study was an open-label two-arm parallel randomized controlled trial. Women (18–75 years) undergoing breast cancer surgery were allocated to either electronic or traditional data collection and completed pain-related questionnaires at baseline, postoperative period, and 3-month follow-up (NCT02240199).

**Results**: We acquired outcome data at all time points from 78 randomized patients, 38 in the electronic group and 40 in the traditional group. The number of data queries (e.g., erroneously entered data) per patient was higher in the electronic data group (4.92 [SD = 4.67] vs. 1.88 [SD = 1.51]; *P* < 0.001). No between-group differences were observed for compliance with medications, data completeness, loss to follow-up, or patient or research assistant satisfaction. More research assistant time per patient was spent collecting data in the traditional group (42.6 min [SD = 12.8] vs. 9.92 min [SD = 7.6]; *P* < 0.001); however, costs per patient were higher in the electronic group ($176.85 [SD = 2.90] vs. $16.33 [SD = 4.90]; *P* < 0.001).

**Conclusion**: Electronic data collection is feasible for perioperative pain clinical trials. Additional trials, including different surgical populations, are needed to confirm our findings and optimize use of electronic data capture methods.

## RÉSUMÉ

**Contexte**: La collecte électronique de données est de plus en plus disponible en tant que moyen de recueillir les données dans le cadre d’essais cliniques liés à la douleur; toutefois, l’efficacité et le coût de cette méthode, comparativement à la collecte de données traditionnelle, sont incertains.

**But**: Évaluer la qualité des données, le respect du protocole, la satisfaction et les ressources requises dans le cadre de la collecte électronique de données (i.e. la transmission électronique par Internet) comparativement aux méthodes de collecte de données traditionnelles (c.-à.-d les cahiers papier et les entrevues téléphoniques) dans un essai randomisé contrôllé factoriel périopératoire.

**Méthodes**: Cette étude était un essai contrôlé randomisé ouvert parallèle à deux volets. Des femmes (de 18 à 75 ans) subissant une chirurgie pour traiter un cancer du sein ont été réparties en deux groupes, soit celui de la collecte életronique de données et celui de la collecte traditionnelle de données. Elles ont ensuite répondu à des questionnaires liés à la douleur au départ, pendant la période post-opératoire et au bout de trois mois (NCT02240199).

**Résutats**: Nous avons receuilli les données de 78 patientes randomisées à chaque point dans le temps : 38 patientes dans le groupe électronique et 40 patientes dans le groupe traditionnel. Le nombre de questions relatives aux données (ex.: données saisies de manière erronée) par patiente était plus élevé dans le groupe de données électroniques (4,92, É.-T. 4,67 comparativement à 1,88, É.-T. 1,51; p < 0,001). Aucune différence n’a été observée entre les groupes en ce qui concerne l’observance du traitement médicamenteux, l’exhaustivité des données, le nombre de patientes perdues de vue et la satisfaction des patientes ou des assistantes de recherche. Les assistants de recherche ont passé plus de temps par patiente pour recueillir les données dans le groupe traditionnel (42,6 minutes É.-T. 12,8 comparativement à 9,92 minutes É.-T. 76; p < 0,001); toutefois, le coût par patiente était plus élevé dans le groupe électronique (176,85 $ É.-T. 2,90 comparativement à 16,33 $ É.-T. 4,90; p < 0,001).

**Conclusion**: La collecte électronique de données est réalisable dans le cadre d’essais cliniques portant sur la douleur périopératoire. Des essais supplémentaires, y compris auprès de différentes populations de patients chirurgicaux, sont nécessaires pour confirmer nos résultats et optimiser l’utilisation de méthodes éectroniques de saisie de données.

## Introduction

Pain is an unpleasant sensory and emotional experience unique to the individual it afflicts. Pain is also a dynamic process, fluctuating both within and between days, and is influenced by mood, emotions, prior experiences, as well as external stimuli such as physical movement.^1–4^ The most common method of measuring pain in clinical and research settings is through the use of self-reported questionnaires, completed by patients verbally or in writing. However, this form of data has issues that may negatively impact the validity of collected data.^5,6^ In particular, this method often requires the patient to recall his or her pain symptoms over a period of time that may range from hours to weeks. The memory of pain is vulnerable to distortion due to physical and psychological contextual factors and the selective coding and retrieval of memories—that is, the memory of pain may be different than the actual experience of pain.^7^

The recall of pain is vulnerable to two known cognitive heuristics, namely, peak–end effect and duration neglect.^7^ *Peak–end effect* is the notion that patients selectively recall intense experiences or those that occur close to the time of reporting. *Duration neglect* means that patients tend to forget the periods of time when they did not experience a phenomenon such as pain. Both of these cognitive biases lead to the overestimation of the severity of the reported pain. For example, in a cohort of patients with rheumatoid arthritis and fibromyalgia, the average intensity of recalled pain as measured on a 100-point visual analogue scale for the previous week was 57, whereas the average of several pain scores taken routinely during the week was 44.^8^ Although short recall periods are often used to minimize recall bias, studies have shown that a recall period as short as even the preceding 24 h may be vulnerable to inaccurate reporting.^9,10^

The momentary assessment of pain (i.e., obtaining pain scores the moment pain occurs) can overcome cognitive biases associated with recalling pain; however, momentary assessments using traditional data collection methods may be problematic. Calling patients at home to obtain daily pain scores is burdensome to both the patient and research team. Paper-based pain diaries can reduce excessive contact while collecting multiple pain scores per day, yet some studies suggest that patient adherence is not optimal and diary entries may be retrospectively fabricated by patients before submission.^11,12^ Furthermore, transferring data from a pain diary to an electronic database for analyses creates the opportunity for data entry errors.

The advent of mobile electronic devices has allowed for novel opportunities to collect patient-reported outcomes for clinical or research purposes. Mounting evidence suggests that data collected via electronic methods may be more accurate and contain fewer errors than traditional methods.^13^ Electronic data capture systems may also aid in improving patient adherence to a clinical trial protocol and satisfaction with reporting.^14–16^ Electronic data capture systems can potentially improve data quality and frequency of reporting, while also decreasing costs associated with data collection.^17^ Though observational data support benefits of electronic data capture systems, there is a paucity of randomized controlled trials comparing electronic to traditional data collection approaches, especially in the perioperative period.^18,19^

Our aim was to characterize the impact of an electronic data collection method on data quality, patient protocol adherence, patient and research assistant satisfaction, and resource requirements in patients undergoing breast cancer surgery when compared to traditional data collection methods (i.e., paper-based diaries and verbally using the telephone).

## Methods

We completed an open-label two-arm parallel randomized controlled trial. This trial was a substudy of the Pregabalin and Lidocaine in breast cancer surgery to Alter Neuropathic Pain (PLAN) pilot trial, which was a multicenter factorial design blinded randomized controlled trial of perioperative pregabalin versus placebo and intraoperative intravenous lidocaine versus placebo in patients undergoing breast cancer surgery. Patients were recruited from Juravinski Hospital in Hamilton, Ontario, and from the Sunnybrook Health Sciences Centre in Toronto, Ontario. Both the pilot trial and this substudy were approved by the ethics review boards at Sunnybrook Health Sciences Centre and Juravinski Hospital, respectively. Before patient enrollment, the pilot trial was registered at clinicaltrials.gov (NCT02240199).

### Patient selection

Eligibility criteria for the substudy included (1) enrollment in the PLAN pilot trial and (2) access to a computer with Internet access at home or a capable smartphone with Internet access (i.e., iPhone 3G or above, iPad first generation or above, LG L7, Sony Xperia Z, Samsung Galaxy III or Nexus, Blackberry Z10, HTC Desire or ONE, Blackberry Q10, Nokia Lumnia 920). Patients enrolled in the PLAN pilot trial were female, 18–75 years old, English-speaking, and undergoing a unilateral or bilateral mastectomy or lumpectomy for prophylaxis or belief of isolated cancerous lesions (biopsy suggestive of cancer or indeterminate) under general anesthesia and provided written informed consent. Patients were excluded if they met any of the following criteria: (1) previous breast surgery within 6 months; (2) undergoing a deep inferior epigastric perforators flap procedure; (3) history of chronic pain or a chronic pain syndrome in the past 3 months; (4) documented hypersensitivity or allergy to pregabalin, gabapentin, or lidocaine; (5) history of ventricular tachycardia, ventricular fibrillation, atrioventricular block type II or III, or congestive heart failure; (6) renal insufficiency (documented creatinine ≥120 μmol/L); (7) known or previously documented cirrhosis; (8) pregnant; (9) unable to swallow study medications; (10) surgeon believes patient is inappropriate for inclusion into trial; (11) unlikely to comply with follow-up (e.g., no fixed address); or (13) patient required gabapentin or pregabalin for a medical condition or has taken these medications daily during the week before randomization.

### Intervention

The electronic data capture system was developed and programmed by InputHealth for specific use in the PLAN pilot trial. Electronic data requests were sent to patients via short message service text message or email according to their preference. In the text message or email, patients received a personalized and encrypted link, which they clicked on to submit electronic data via the Internet. The electronic data capture system abided by privacy regulations in accordance with the Province of Ontario’s Personal Health Information Protection Act, including strong password policy, authorized log-in and two-factor authorization, servers that utilized anti-sniff/anti-spoof firewall defenses with advanced 24/7 monitoring and multilevel intrusion prevention, and anti-spam, anti-malware, and anti-virus implementations. All electronic data were stored in Canada, online interactions were encrypted using 256-bit SSL technology, and stored data were encrypted using AES-256-CBC technology. Patient data collected from the electronic InputHealth system were stored in a third-party database and, at the end of the study, these data were manually transcribed into the central trial database by a research assistant. The electronic platform was piloted internally with simulated patients prior to enrolling study patients. Field validation errors were implemented to reduce inappropriate (i.e., cannot submit alphabetic characters for a response requiring numeric characters) or missing (i.e., cannot submit questionnaire unless all items are completed) responses.

### Control

Data from patients in the control group were collected using traditional data collection methods (i.e., paper-based or verbal) as described in the procedures below. Study personnel manually inputted all of the data into the database.

### Procedures

Patients who were potentially eligible for the PLAN pilot trial were approached at their surgeon’s office or pre-assessment clinic before their operation. Patients who met eligibility criteria and consented to enroll in the PLAN pilot trial were subsequently approached about the electronic data capture substudy. If patients met substudy eligibility criteria and provided written informed consent specific to substudy inclusion, they were randomized (1:1) to the electronic or traditional data collection groups using a centrally controlled, secure Internet-based randomization service to ensure allocation concealment.

We assessed outcomes at baseline, over 9 days postoperative, and at 3-month follow-up. At baseline, patients completed the 0- to 10-point Numeric Rating Scale (NRS) for pain, Pain Catastrophizing Scale, Amsterdam Preoperative Anxiety and Information Scale, and 27-item Somatic Pre-occupation and Coping scale. Patients in the electronic data capture arm completed these questionnaires in hospital or at home. If patients were in hospital, they completed these questionnaires on an iPad that was provided to them. If they were at home, they were sent the questionnaires via email or through a text message if they had a smartphone. Patients in the control group completed baseline questionnaires on paper in person immediately after providing informed consent.

Data collected after surgery included twice-daily NRS pain scores, once-daily opioid and nonopioid pain medication use reports, and once-daily adverse events reports on postoperative days 1 to 3 and 9. No data were collected on postoperative days 4–8. The overall number of data points collected in the electronic and traditional groups were the same. Patients in the electronic data capture arm were sent an email or text message twice a day on postoperative days 1, 2, 3, and 9 to complete an electronic version of the NRS for pain. For the morning pain score, patients received an electronic data request at 8:00 am and were asked to complete the request by noon. For the evening pain score, patients were sent a request at 8:00 pm and asked to complete the request by midnight. Both morning and evening data requests included a reminder for the patient to take daily study medications, and the evening data request also collected data on opioid and nonopioid medication use and adverse events. If the patient did not complete the morning and evening data request by 11:00 am or 11:00 pm, respectively, another electronic reminder was sent. If no data was received by the patient 1 h after the reminder, an electronic notification was sent to the research team to follow up with the patient. On postoperative days 4–8 in the electronic arm, text messages were sent to patients to remind them to take the study medication.

Patients in the control group completed postoperative data using a paper pain diary that was provided to them after surgery. On postoperative days 1 to 3 and 9, these patients were asked to record their NRS pain scores in the morning and evening, medication use, study drug compliance, and adverse events in a paper diary. Research personnel called patients in the control group at home on postoperative days 1, 4, and 10 to obtain their data. There were no reminders in the control group during postoperative days 4–8.

At 3 months after surgery, data on the primary outcomes of the pilot trial, as well as data from the Short-Form McGill Pain Questionnaire 2 (scored by averaging 22 items of pain intensities rated on a 0–10 scale, with higher scores indicating more pain), Brief Pain Inventory (composite of pain severity and interference scores on a 0–10 scale, with higher scores indicating worse severity or interference), and the 36-item Short Form Survey (weighted sums of scores across eight domains transformed into a 0–100 scale, with higher scores denoting more disability) were collected. In the electronic data capture arm, patients were sent an email or text message to complete these questionnaires. In the traditional data collection group, patients were called at home and verbally provided responses to a research assistant. If patients in the electronic data arm did not complete the data request within a week, a research assistant called them to gather data over the telephone.

### Outcomes

Our primary outcome was data quality, evaluated by determining the average number of data queries (and the associated standard deviation [SD]) in the electronic data collection group and traditional data collection group. Queries are concerns about a specific data point raised by the data manager or study coordinator. These queries are raised because data seem inappropriate (i.e., field asks for a three-digit number but only one digit is recorded), contain an error or are missing, incongruent for the specified field (i.e., recorded data are outside possible data range), or simply need further clarification (e.g., data entered are accurate but seem abnormal). Queries requiring intervention more likely denote that data were entered erroneously because research assistants had to go back and change previously entered data. Queries were posted by the study coordinator who was blind to patient group allocation for the pilot trial and substudy.

Secondary outcomes included protocol adherence, patient satisfaction, and an economic analysis. Protocol adherence was defined as the proportion of patients who self-reported taking study medications as directed during the study period. This outcome related only to compliance with perioperative pregabalin because the intraoperative intravenous lidocaine infusion was administered by an attending anesthesiologist. We reported loss to follow-up by recording the proportion of patients who remained in the study until completion (i.e., the total number of patients randomized minus those lost to follow-up and withdrawals at 3 months after surgery).

We asked patients to rate their satisfaction with respect to their data collection method on a scale of 0 to 100 at 3-month follow-up, with 0 being *not satisfied* and 100 being *extremely satisfied*. Similarly, research assistants rated their satisfaction with each data collection method at the end of the study on the same 0 to 100 scale.

Finally, an economic analysis was conducted. The time that a research assistant spent collecting patient data was recorded in minutes throughout the study and included any time spent troubleshooting technical difficulties with the electronic system. A cost-effective analysis was performed by comparing the average cost of the electronic data capture system to traditional data collection methods. Research assistants’ time was included in the economic analysis by determining the time (in hours) they spent performing data collection for all patients multiplied by an hourly wage of C$23. The cost associated with an electronic data capture system included third-party (InputHealth) platform development and service fee, iPads, and other incidental fees. These costs were combined with research assistant time per hourly salary to determine total cost of an electronic data collection platform. The costs associated with traditional data collection were research assistant time per hourly salary.

### Sample size

An internal analysis of a similar pilot randomized controlled trial conducted at the Population Health Research Institute at McMaster University showed the average number of queries per patient to be 25 with a standard deviation of 5. With an alpha of 0.05 and 80% power, we required at least 32 patients in total to identify a reduction in queries from a mean of 25 to a mean of 20. Our trial was powered around detecting differences in data queries because of the importance of collecting high-quality patient-reported outcome data in pain research and clinical settings.^20,21^

### Statistical analysis

Though group allocation was revealed to patients, research assistants, and clinical care providers as a consequence of the interventions, data analysts remained blinded until completion of analyses. Continuous outcomes such as total queries, queries requiring intervention, queries for missing data, patient and research assistant satisfaction, research assistant time, and costs (in Canadian dollars) were analyzed using *t* tests or Wilcoxon nonparametric tests if data were nonnormal. Outcomes of proportions such as drug compliance and patient retention were analyzed using Fisher’s exact test due to small sample sizes. All statistical analyses were performed using SPSS software version 20 (Armonk, NY). All tests were two-sided and significance was considered at *P* < 0.05.

## Results

**Figure 1.**
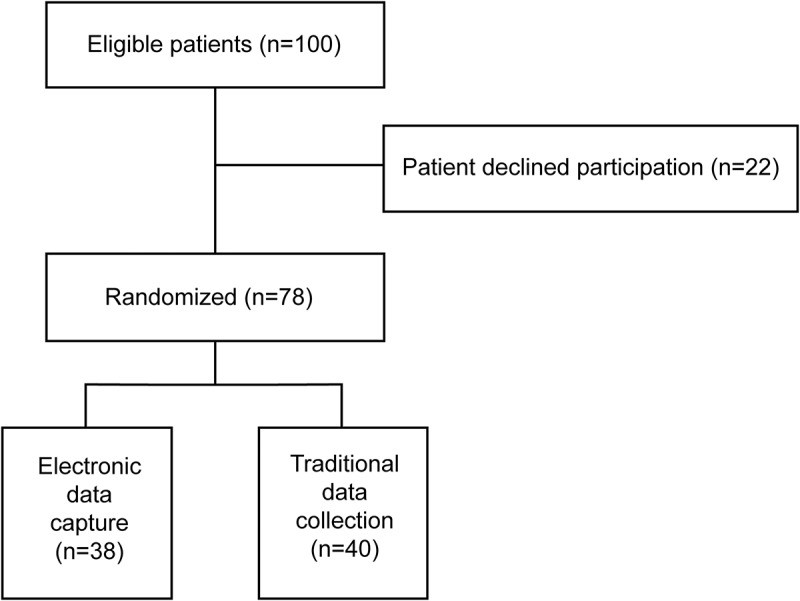
Patient flow diagram.

Of the 100 patients included in the PLAN pilot trial, 22 patients declined participation in the electronic data capture substudy. A total of 78 patients provided informed consent and were randomized, 40 to traditional data collection and 38 to electronic data collection (Figure 1). Females included in this trial were on average of 53 (SD = 11) years old. Most patients were of European ethnicity (90%), married (78%), had completed college or university (73%), and were engaged in full-time employment (62%; Table 1).

**Table 1.** Baseline characteristics.

	Electronic data collection	Traditional data collection	Overall
	*n* = 38	*n* = 40	*n* = 78
Age (years), mean (SD)	52.3 (12.2)	53.1 (10.1)	52.7 (11.1)
Height (cm), mean (SD)	164.4 (6.4)	165.6 (7.3)	165.0 (6.9)
Weight (kg), mean(SD)	78.0 (21.1)	76.9 (17.8)	77.4 (19.4)
Reason for surgery, *n* (%)			
Breast cancer/belief of cancerous lesions	35 (92.1)	38 (95.0)	73 (93.6)
Prophylactic surgery	3 (7.9)	2 (5.0)	5 (6.4)
Ethnicity, *n* (%)			
European	35 (92.1)	35 (87.5)	70 (89.7)
Asian	0 (0.0)	2 (5.0)	2 (2.6)
Middle Eastern	1 (2.6)	0 (0.0)	1 (1.3)
Native/Aboriginal	1 (2.6)	0 (0.0)	1 (1.3)
Black African or black Caribbean	0 (0.0)	0 (0.0)	0 (0.0)
Other	1 (2.6)	3 (7.5)	4 (5.1)
Married/common law, *n* (%)	31 (81.6)	30 (75.0)	61 (78.2)
Education, *n* (%)			
University degree or more	15 (39.5)	14 (35.0)	29 (37.2)
College diploma	11 (28.9)	17 (42.5)	28 (35.9)
High school	10 (26.3)	9 (22.5)	19 (24.4)
Less than high school	2 (5.3)	0 (0.0)	2 (2.6)
Employment status, *n* (%)			
Employed, full-time	21 (55.3)	27 (67.5)	48 (61.5)
Unemployed	11 (28.9)	10 (25.0)	21 (26.9)
Current smoker, *n* (%)	2 (5.3)	4 (10.0)	6 (7.7)
Randomized to active lidocaine, *n* (%)	18 (47.4)	21 (52.5)	39 (50.0)
Randomized to active pregabalin, *n* (%)	17 (44.7)	21 (52.5)	38 (48.7)

### Data quality

Although the average number of data queries in both groups was low, patients in the electronic data capture group had a significant increase in the total number of queries (4.92 [SD = 4.67] vs. 1.88 [SD = 1.51]; *P* < 0.001) and queries requiring intervention (3.42 [SD = 3.63] vs. 1.23 [SD = 1.29]; *P* < 0.001) compared to the traditional data capture group. There were no differences in the percentage of queries requiring intervention to total queries (69.5 vs. 65.4; *P* = 0.936) and the number of queries related to missing data between the two groups (1.53 [SD = 2.70] vs. 0.90 [SD = 0.87]; *P* = 0.56; Table 2).

**Table 2.** Data quality outcomes.

	Electronic data collection	Traditional data collection	
	*n* = 38	*n* = 40	*P* value
Average number of queries per person (SD)	4.92 (4.67)	1.88 (1.51)	<0.001
Average number of queries requiring intervention per person (SD)	3.42 (3.63)	1.23 (1.29)	<0.001
Proportion of queries requiring intervention per total queries (%)	69.5	65.4	0.936
Average number of questionnaires with at least one field marked missing per person (SD)	1.53 (2.70)	0.90 (0.87)	0.563

### Protocol adherence

There were no differences between the two data collection groups in patient-reported compliance with study medications during the nine postoperative days (*P* value for difference ranged from 0.15 to 0.97; Table 3). Further, there were no differences between groups on numeric pain score reporting (*P* value for difference ranged from 0.23 to 1.00) or any differences in the number of patients lost to follow-up (*P* value for difference ranged from 0.49 to 01.00; Supplementary Data Tables 1 and 2).

**Table 3.** Patient-reported compliance with postoperative study medications.

	Electronic data capture	Traditional data capture	
	*n* = 38	*n* = 40	*P* value
Preoperative dose	38 (100)	39 (97.5)	1.000
Postoperative day 1	36 (94.7)	39 (97.5)	0.610
Postoperative day 2	37 (97.4)	39 (97.5)	1.000
Postoperative day 3	36 (94.7)	39 (97.5)	0.610
Postoperative day 4	36 (94.7)	40 (100)	0.234
Postoperative day 5	37 (97.4)	40 (100)	0.487
Postoperative day 6	34 (89.5)	39 (97.5)	0.195
Postoperative day 7	35 (92.1)	39 (97.5)	0.352
Postoperative day 8	34 (89.5)	38 (95.0)	0.425
Postoperative day 9	35 (92.1)	38 (95.0)	0.671

### Satisfaction

**Figure 2.**
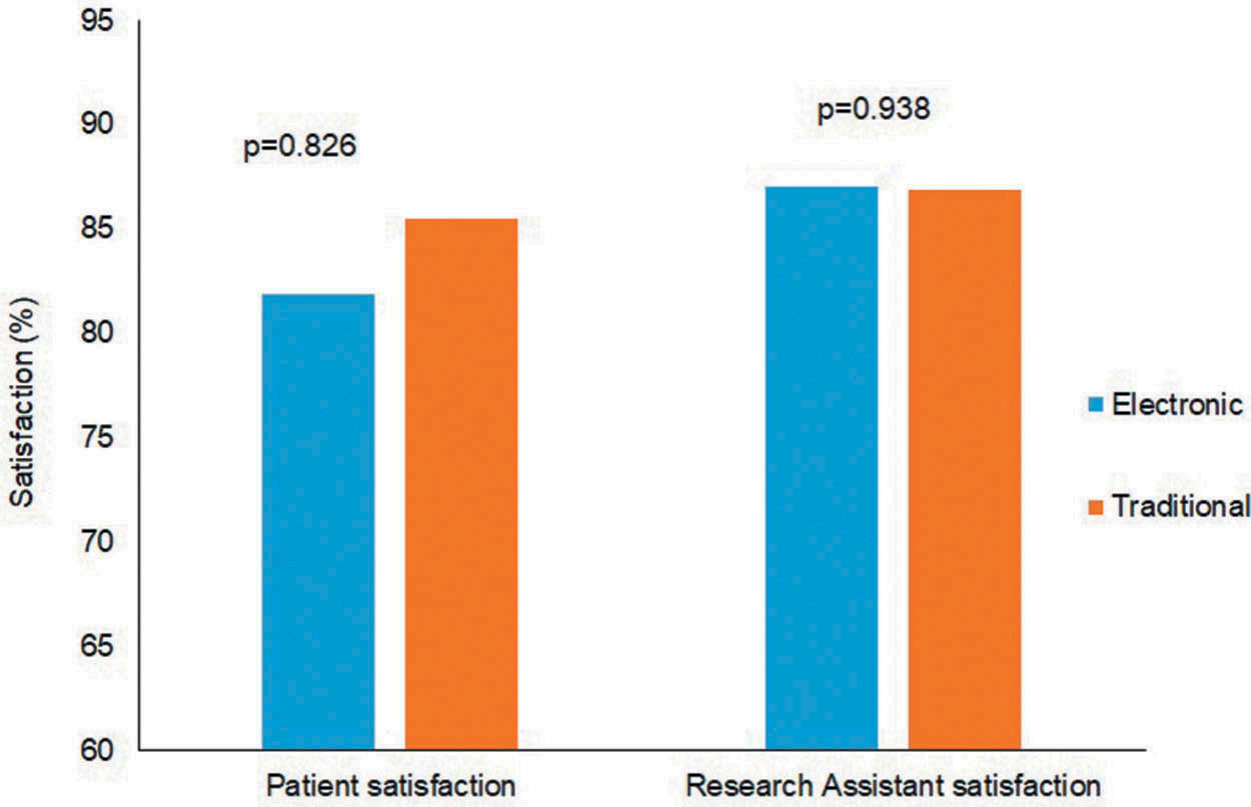
Patient and research assistant satisfaction.

There were no differences in patient satisfaction scores between the electronic data capture group (81.9 [SD = 28.6]) and traditional data collection group (85.5 [SD = 22.1]; *P* = 0.83; Figure 2). Similarly, at the end of the study, there was no difference in research assistant satisfaction scores across groups (87.0 [SD = 5.1] for electronic data collection group vs. 86.9 [SD = 5.0] for traditional data collection group; *P* = 0.94).

### Economic analysis

Table 4 depicts the average research assistant time spent in minutes at each visit with patients in the electronic and traditional data collection groups during the study. Time in minutes with patients in the electronic data collection group was significantly less than that with the traditional group at baseline (8.53 [SD = 6.6] vs. 11.28 [SD = 4.9]; *P* = 0.02), postoperative day 3 (2.08 [SD = 2.5] vs. 6.83 [SD = 3.7]; *P* < 0.001), postoperative day 9 (1.33 [SD = 0.7] vs. 4.15 [SD = 2.2]; *P* < 0.001), and 3-month follow-up (5.67 [SD = 4.0] vs. 17.53 [SD = 8.7]; *P* = 0.02). The average time required per patient was 9.92 min (SD = 7.58) in the electronic group and 42.60 min (SD = 12.78; *P* < 0.001) in the traditional group.

**Table 4.** Study personnel time and costs per patient.

	Electronic data capture	Traditional data capture	
	*n* = 38	*n* = 40	*P* value
Mean time in minutes (SD)			
Baseline	8.5 (6.6)	11.3 (4.9)	0.024
Postoperative day 1	2.7 (1.5)	2.8 (1.8)	0.947
Postoperative day 3	2.1 (2.5)	6.8 (3.7)	<0.001
Postoperative day 9	1.3 (0.7)	4.2 (2.2)	<0.001
3-month follow-up	5.7 (4.0)	17.5 (8.7)	0.016
Total time spent per patient	9.9 (7.6)	42.6 (12.8)	<0.001
Total time spent (min)	377	1704	—
Total costs per patient ($)	176.9 (2.90)	16.3 (4.90)	<0.001

When including the salary costs associated with research assistant time, as well as the start-up costs required for the electronic data capture system (i.e., third-party platform, iPads), the total costs per patient were higher in the electronic group ($176.85 [SD = 2.90] vs. $16.33 [SD = 4.90]; *P* < 0.001; Table 4).

## Discussion

Our study found that electronic collection of pain data, versus traditional methods, resulted in less time required by research assistants when patients provided their data electronically; however, there was an increase in data queries. Mode of data collection did not affect patient compliance, loss to follow-up, or satisfaction. The economics of electronic data capture may be more favorable in large trials.

Previous reports have suggested that electronic data capture systems may improve data quality in clinical studies.^22^ Platforms can be built such that they will not accept missing or incongruent responses to study questionnaires, whereas the use of paper-based questionnaires creates difficulties for readily assessing and correcting missed or erroneous responses. In our trial we observed a significant increase in the total number of data queries and data queries requiring intervention with the electronic data capture compared to traditional methods. Overall, the number of data queries per person across both groups was, however, low given the number of data collection points and patient reported outcome items to be completed (i.e., average of fewer than five queries per person in this study).

The increased number of data queries in the electronic data collection group can partially be explained by the possibility of a transcription error and limited piloting. In particular, data collected by research assistants via traditional methods were entered directly into our central trial database, whereas data for patients in the electronic group were collected and stored in a separate database. At the end of the study, electronically collected data were manually transcribed into the central trial database, introducing the possibility of a transcription error. Further, upon reviewing sources of queries, many queries in the electronic group arose from postoperative opioid consumption data. Patients often reported the incorrect units for reported medications (i.e., reported unit in milligrams for the number of Perocets used). We initially piloted the electronic platform internally with simulated patients and further piloting with enrolled patients would have likely identified potential sources of errors before implementing the platform.

Though the electronic data capture system was associated with an increase in data queries, the electronic system captured 97%–100% of postoperative pain scores within the specified 4-h window (Supplementary Appendix Table 1). Paper diaries were given to patients in the traditional group to record their respective pain scores; however, we have no certainty that their data were reported during those specified times. Prior studies suggest that patients are notoriously noncompliant with completing a symptom diary and will tend to quickly fill in missing values prior to submission.^11^ Timestamping features of an electronic data collection system allow investigators to know the exact time data was recorded, which is critically important for momentary assessments. Furthermore, electronic data capture systems can allow for multiple assessments without an increased burden to patients or investigators.^7^ Previous studies suggest that patients can be prompted for responses up to six times a day without any significant burden or interference.^8^

Previous reports in clinical care have documented improved medication adherence with the use of electronic reminders,^23^ yet few have evaluated their effect on study medication compliance in a clinical trial. Our electronic platform combined with text message reminders did not have any effect on patient compliance with study medications. The already high compliance rate (close to 100%) in the traditional data collection group left little room for improvement. We also observed no differences in attempts to complete questionnaires at each data collection point or patients who were lost to follow-up. These findings again may be indicative of a highly engaged study cohort.

Our findings of no difference in patient satisfaction may be related to lack of exposure to the alternate data collection method; a phenomenon that has been observed in another study assessing satisfaction with electronic versus paper-based pain questionnaires.^24^ The ideal study design to evaluate patient satisfaction and preference would be a crossover randomized trial, where all patients would have an opportunity to trial both methods. Indeed, several studies that have used a crossover design have shown electronic pain reporting to be preferable to patients.^15,25,26^ Nonetheless, research assistants, who were exposed to both methods, did not appear to have a preference and reported high satisfaction with both data collection methods.

The use of the electronic data capture system in this study was associated with an increase in financial costs of approximately C$6000 over traditional data collection methods. However, most of these costs were related to creating and developing the platform and, as such, are fixed and not influenced by the number of patients enrolled. In contrast, research assistant time and associated cost are directly proportional to the size of the study. Given that electronic data capture systems have primarily fixed costs yet reduce research assistant time, the larger a clinical trial, the more likely an electronic platform would become cost-effective. Figure 1 in the Supplementary Data presents an extrapolation of the costs of each data collection method per number of patients in our study. The figure shows that for a trial with more than 400 patients, an electronic data capture system becomes cost effective. Though these projections are based on the costs associated with the third-party vendor used in our trial, they demonstrate that the benefit of reducing research assistant time becomes more pronounced as the number of participants in a study increases. Two additional cost and resource considerations in using an electronic data collection system relate to obtaining a license in using certain instruments or patient-reported outcomes in an electronic form and start-up costs with developing a third-party platform. Our study investigators spent several weeks working with a third-party vendor to design, develop, pilot, and test a custom-built platform for this study. Because this time was donated in-kind it is not reflected in our economic analysis. Investigators looking to use an electronic platform should consider this additional time and resource cost.

There are several limitations to this investigation. First, this was an open-label study, and though participant blinding was not possible, this could introduce bias; however, nonsignificant differences in satisfaction scores would suggest minimal bias from the lack of blinding. Second, although the external validity of our results is bolstered by participant recruitment and data collection across two clinical sites and an electronic platform that integrates with all mobile smart devices, our results are not generalizable to all electronic data capture systems. In particular, other systems may differ significantly from that used in this study in terms of their functionalities, user interfaces, security features, mobile and Web-based integrations, and costs. Third, our event rate was less than expected (i.e., approximately five data queries per patient, whereas our sample size calculation was based on 25 queries per patient), rendering our study underpowered. Lastly, due to the nesting of this study within the PLAN pilot trial, we were unable to use a randomized crossover design to compare the electronic and traditional data collection methods. This limited our ability to examine within-patient differences in outcomes such as questionnaire completion and satisfaction. Finally, for our time and resource analyses, we were not able to collect data on how long research assistants spent transcribing data for patients in the traditional data collection group into the central trial database and, similarly, transcribing data for electronically collected data to the central trial database.

Overall, our study suggests that electronic data capture systems are a viable alternative to collect data in a perioperative pain clinical trial. These systems can also significantly reduce the amount of time needed to collect study data, and this reduction in research assistant time can be cost-saving for studies of 400 or more patients. Future studies with larger sample sizes and crossover designs are needed to verify results of our study and to further document the benefits and limitations of using electronic data collection systems in pain trials.

## Supplementary Material

Supplemental Material
