# Supplementary material for: Electronic Versus Traditional Data Collection: A Multicenter Randomized Controlled Perioperative Pain Trial
Source: Can J Pain. 2019 Jul 30;3(2):16–25. doi: 10.1080/24740527.2019.1587584 (PMC8730625; doi:10.1080/24740527.2019.1587584)
Supplement: Supplemental Material [file UCJP_A_1587584_SM8163.docx]

**Supplementary Appendix**

**Figure 1: Costs of data collection method per study size**
